# Design and *In Vitro* Evaluation of a New Nano-Microparticulate System for Enhanced Aqueous-Phase Solubility of Curcumin

**DOI:** 10.1155/2013/724763

**Published:** 2013-07-28

**Authors:** Diana Guzman-Villanueva, Ibrahim M. El-Sherbiny, Dea Herrera-Ruiz, Hugh D. C. Smyth

**Affiliations:** ^1^Division of Pharmaceutics, College of Pharmacy, The University of Texas at Austin, TX 78712, USA; ^2^Facultad de Farmacia, Universidad Autónoma del Estado de Morelos, 62209 Cuernavaca, MOR, Mexico; ^3^Polymer Laboratory, Faculty of Science, Mansoura University, Mansoura 35516, Egypt; ^4^Center for Materials Science, Zewail University, Zewail City of Science and Technology, 6th of October City, Giza 12588, Egypt

## Abstract

Curcumin, a yellow polyphenol derived from the turmeric *Curcuma longa*, has been associated with a diverse therapeutic potential including anti-inflammatory, antioxidant, antiviral, and anticancer properties. However, the poor aqueous solubility and low bioavailability of curcumin have limited its potential when administrated orally. In this study, curcumin was encapsulated in a series of novel nano-microparticulate systems developed to improve its aqueous solubility and stability. The nano-microparticulate systems are based entirely on biocompatible, biodegradable, and edible polymers including chitosan, alginate, and carrageenan. The particles were synthesized via ionotropic gelation. Encapsulating the curcumin into the hydrogel nanoparticles yielded a homogenous curcumin dispersion in aqueous solution compared to the free form of curcumin. Also, the *in vitro* release profile showed up to 95% release of curcumin from the developed nano-microparticulate systems after 9 hours in PBS at pH 7.4 when freeze-dried particles were used.

## 1. Introduction

Curcumin, a natural yellow polyphenol present in the root of *Curcuma longa*, has been commonly used in the food industry as a food spice and coloring agent [1, 2]. It has been demonstrated that curcumin possesses multiple pharmacological activities including anti-inflammatory, antioxidant, antihyperlipidemic, antiulcer, immunomodulatory, neuroprotective, antineoplastic, and chemoprotective properties [3–8]. These therapeutic characteristics are mediated via downregulation of transcription factors such as nuclear factor (NF-*κ*B), beta-catenin, hypoxia inducible factor (HIF)-1*α*, and cyclo-oxygenase-2 and the upregulation of PPAR-*γ*, superoxide dismutase, and caspases [9–13]. Curcumin safety, tolerability, and low toxicity have been well established in human trials [14–16].

In spite of the diversity of bioactivities of curcumin and its potential to prevent and treat diseases, its limited aqueous solubility and instability in the gastrointestinal tract yield poor oral absorption, low bioavailability [1, 5, 9, 17], and poor therapeutic performance [18–20]. Low serum levels as well as limited tissue distribution and rapid metabolism have been reported when curcumin is administrated [18].

Formulations based on liposomes, micelles, microemulsions, nanocrystal and amorphous solid dispersions, and gastroretentive floating systems have been developed to improve the curcumin oral bioavailability [17–23]. However, a considerable interest has recently been paid to the development of polymeric nanoparticles-based drug delivery systems [5, 23–26] due to these systems ability to overcome several problems related to the delivery of hydrophobic drugs dispersed in aqueous solutions.

Polymers derived from natural sources have been used extensively in the pharmaceutical and food industry. Among these polymers, polysaccharides have been widely utilized because of their biocompatibility, biodegradability, and low toxicity. Chitosan, a natural polymer obtained from the deacetylation of chitin, has been a useful polymer for various biomedical applications, sustained drug release and for enhancement of bioavailability of poor water-soluble drugs. Its wide range of applications makes this polymer an attractive candidate for oral drug and supplement delivery applications [27–31].

Alginate, a linear polysaccharide consisting of *α*-L-guluronic acid (G) and *β*-D-mannuronic acid (M) residues joined together in blocks, is commonly used due to its mucoadhesive properties and its ability to form three-dimensional hydrogel networks. The ability of alginate to form hydrogels is attributed to the capacity of its guluronic acid residues to bind with multivalent cations such as Ca^2+^ through ionic interactions [32, 33]. The alginate-based hydrogel networks have been found very efficient in entrapment and controlled delivery of a variety of ingredients including drugs, proteins, bacteria, enzymes, and cells [34–36].

Carrageenan is another important hydrophilic polysaccharide composed of D-galactose and 3,6-anhydro-D-galactose units. According to the number and position of sulfate groups, carrageenans are classified as kappa (*κ*), iota (*γ*), alpha (*α*), beta (*β*), lambda (*λ*), and theta (*θ*) [33]. Carrageenan is commonly used in food products as a thickening and stabilizing agent, and it has been recently introduced to pharmaceutical formulations for controlled release purposes due to its gelling ability [37–39]. Under appropriate conditions, the gelation of *κ*-carrageenan occurs in the presence of potassium ions forming strong gels that can be used as drug carriers [28].

The purpose of this study was to develop and evaluate curcumin-loaded nano-microparticulate systems to improve the solubility and stability of curcumin in gastrointestinal (GI) conditions. These nano-microparticles carriers are based on naturally occurring polymers including chitosan, carrageenan, and alginate which have desirable characteristics such as biocompatibility, biodegradability, and nontoxicity. 

Encapsulation of the curcumin-loaded chitosan nanoparticles into alginate-carrageenan matrices would confer protection and stability to curcumin during its transit along the GI tract, thus increasing the amount of curcumin available to exert its pharmacological effect. Moreover, the presence of alginate in the developed hydrogel matrices allows sustained release of curcumin as the particles pass down the GI tract [40, 41]. In addition, this study reports the effect of polymer composition and drying method on the surface morphology and size of the developed nano-microcarriers, as well as on the release behavior of curcumin from the developed nano-microparticle systems.

## 2. Experimental

### 2.1. Materials

Curcumin from *Curcuma longa* (turmeric), Chitosan (medium MW, % N-deacetylation about 76.4%, as determined by elemental analysis), sodium alginate (low viscosity, 250 cps for 2% w/v solution at 25°C), carrageenan of commercial grade type I, sodium triphosphate pentabasic (TPP) of practical grade 90–95%, and potassium chloride were obtained from Sigma Aldrich (St. Louis, MO). Polyvinyl alcohol (PVA), 99-100% hydrolyzed, MW 86000, was obtained from Acros organics (New Jersey, USA); anhydrous calcium chloride was purchased from EMD Chemicals Inc. (Darmstadt, Germany). Phosphate-buffered saline (PBS, pH 2.1 and 7.4) and all other reagents were of analytical grade and used as received.

### 2.2. Methods

#### 2.2.1. Preparation of Curcumin-Loaded Chitosan Nanoparticles

The curcumin-loaded chitosan nanoparticles were prepared using the ionotropic gelation technique. Briefly, an aqueous chitosan solution (0.05% w/v) was prepared using 5 mL of 0.06 M acetic acid and made up to a 500 mL with distilled water. Then, 50 *μ*L of 2% (w/v) aqueous PVA solution and 80 mL of 0.1% TPP solution were mixed and added to 200 mL of chitosan solution. The final mixture was sonicated using an ultrasonic processor (Misonixsonicator 4000, NY, USA) for 4 min with dropwise addition of the alcoholic curcumin solution (0.5 g of curcumin dissolved in 15 mL of ethanol) to obtain a final concentration of 1.7 mg/mL of curcumin. The curcumin-loaded chitosan nanoparticle dispersion was then kept in a fume hood under stirring to allow ethanol evaporation and then used in the next step. 

#### 2.2.2. Preparation of Alginate-Carrageenan Hydrogel Microparticles

Different hydrogel matrices based on sodium alginate and carrageenan containing curcumin-loaded chitosan nanoparticles were developed via ionotropic gelation using a crosslinking mixture of calcium and potassium chloride solutions (Table 1). Briefly, 20 mL of curcumin-loaded chitosan nanoparticle suspension prepared in Section 2.2.1 was added dropwise while stirring to alginate-carrageenan solutions of different compositions maintaining the final polymer concentration constant (2% w/v) in all formulations (Table 1). Each polymer mixture was extruded while stirring in 80 mL (1 : 1, 3% w/v) of a calcium chloride and potassium chloride solution using a syringe with a 21-gauge needle. The formed microparticles were kept for 15 min in the crosslinkers solution with slow stirring to complete the crosslinking. Finally, the crosslinked microparticles were collected and washed with distilled water (20 mL) to remove the unreacted polymers. The obtained microparticles were fractioned and either freeze-dried or air-dried at room temperature and then stored for further analysis. 

#### 2.2.3. Particle Size Measurements

The size of the curcumin-loaded chitosan nanoparticles was determined by SEM (scanning electron microscopy, Zeiss Supra 40 VP) with the aid of the ImageJ software 1.44o version (NIH). The diameters of the hydrogel microparticles were measured using a digital micrometer (General Ultra-Tech).

#### 2.2.4. Particle Morphology Characterization

The morphology of the developed hydrogel microparticles was investigated by scanning electron microscopy (SEM, Zeiss Supra 40 VP). The dried particles were mounted on aluminum stubs and coated with a 50/50 mixture of Au/Pd and then scanned at a 10 kV.

#### 2.2.5. Swelling Behavior of the Hydrogel Microparticles

The swelling behavior of the freeze-dried and air-dried hydrogel nano-microparticles was studied by incubating certain amount of particles (10–15 mg) in an Eppendorf tube containing 1.5 mL of PBS (pH 2.1 or 7.4) with shaking at 100 rpm at 37°C. At predetermined intervals (1 and 2 h incubation at pH 2.1 and 3, 4, 5, 6, 7, 8 up to 24 h incubation at pH 7.4), swollen samples were removed from the PBS solution and weighted after blotting their surface to calculate their swelling percentages using the following relationship [42].

(1) $$\begin{array}{c} S\;\left(\%\right)=\frac{W_{t}-W_{0}}{W_{0}}\times 100, \end{array}$$

where *W*_0_ is the initial weight and *W*_*t*_ is the weight of the swollen particles at time *t*. The swelling data represents the average ± SD from three independent swelling experiments.

#### 2.2.6. Release Study

The cumulative release of curcumin from the developed hydrogel nano-microparticles was carried out through incubating preweighed samples (10–15 mg) while shaking at 100 rpm in 1 mL of buffer solutions at pH 2.1 (1 and 2 h incubation) or at pH 7.4 (3, 4, 5, 6, 7, 8 up to 24 h incubation) at 37°C. After incubation, 100 *μ*L aliquots were removed from the buffer solution to be analyzed by UV-Vis spectrophotometry (Infinite M 200 fluorophotometer, TECAN, USA) at *λ*_max⁡_ of 430 nm. The withdrawn sample was replaced with equal volume of fresh buffer solution to keep the volume of release fluid constant. The collected data represents the mean ± SD from three independent release experiments.

#### 2.2.7. Statistical Analysis

All the data was obtained from three independent experiments analyzed and expressed as mean ± SD.

## 3. Results and Discussion

### 3.1. Preparation of Curcumin-Loaded Chitosan Nanoparticles

**Figure 1:**
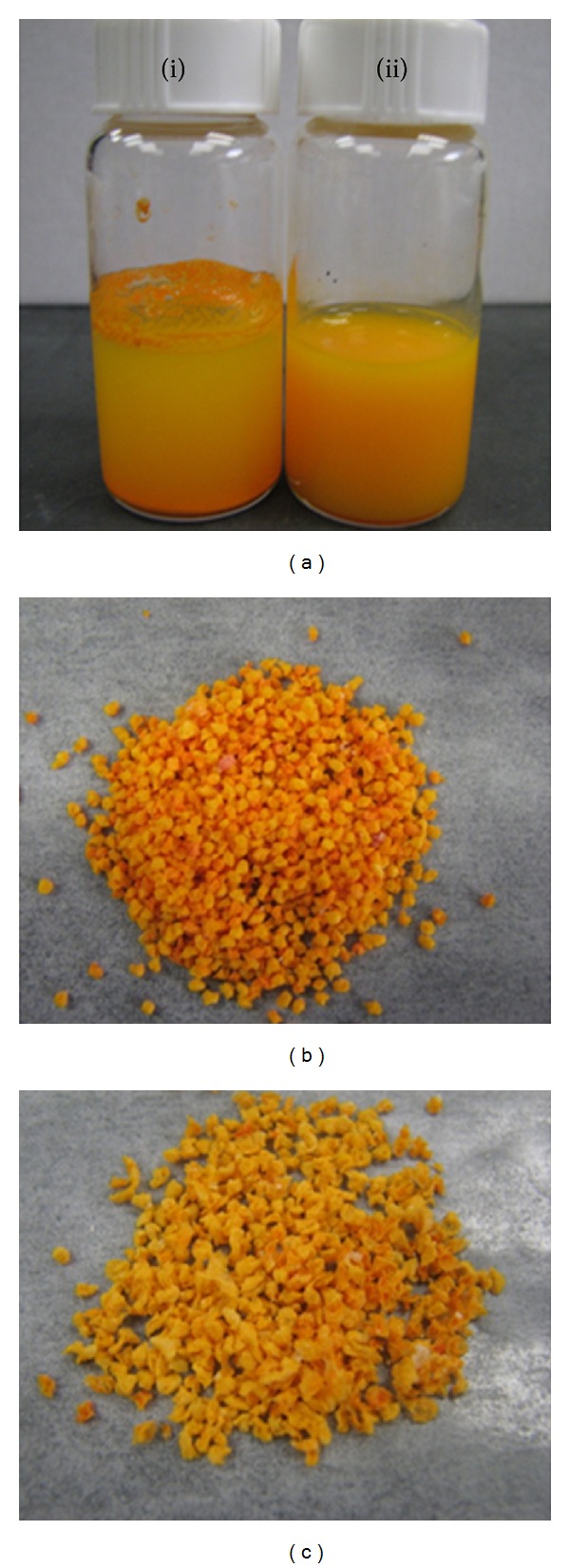
Photographs showing (a) (i) curcumin suspended in distilled water and (ii) aqueous suspension of curcumin-loaded chitosan nanoparticles, and microscopic images showing the effect of composition on the general shape of the developed curcumin-loaded hydrogel microparticles; (b) microparticles based on alginate alone and (c) microparticles composed of (50 : 50) alginate and carrageenan.

**Scheme 1:**
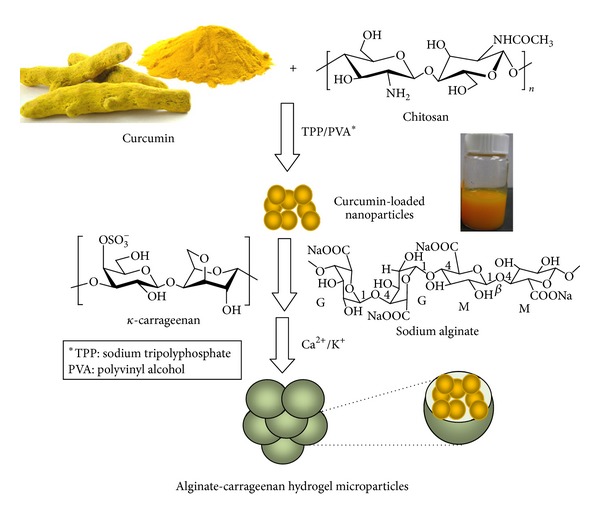
Schematic illustration of the developed curcumin-loaded nano-microparticles.

Chitosan-based nanoparticles were prepared by the ionotropic gelation method and investigated as potential carriers that could enhance the solubility and bioavailability of curcumin (Scheme 1). As can be noted from Figure 1(a)-(i), the free curcumin (100 mg/mL) formed a large flocculate on the surface due to its very limited solubility in water (0.6 *μ*g/mL) [43]. In contrast, the curcumin encapsulated into chitosan nanoparticles was homogenously dispersed in water at a higher concentration (180 mg/mL) (see Figure 1(a)-(ii)). Furthermore, the curcumin nanosuspension displayed the natural intense yellow color from curcumin, which could be an indicative of the curcumin incorporation into the nanoparticle dispersion. In contrast, free curcumin was not incorporated into the aqueous environment, and most of it was precipitated. Thus, the encapsulation of curcumin into chitosan nanoparticles may be beneficial in improving the curcumin concentration and enhancing its dispersity in aqueous solution, which consequently may improve curcumin dissolution and the resulting bioavailability when administrated orally.

### 3.2. Preparation of Alginate-Carrageenan Hydrogel Microparticles

Alginate-carrageenan hydrogel microparticles were obtained via ionotropic gelation of a mutual aqueous mixture of alginate and carrageenan through interactions with their counter ions (Ca^2+^ and K^+^ ions, resp.) as shown in Scheme 1. 

During the preparation of the hydrogel matrices, it was microscopically observed that stable microparticles were instantaneously crosslinked and formed when only alginate was present (100%, F1 formulation). Conversely, for formulations containing carrageenan (30%–50%, F2 and F3), the gelation process took place more slowly depending on the carrageenan content in each formulation, leading to the formation of less spherical matrices compared with the microparticles based on alginate alone (Figures 1(b) and 1(c)). This may be attributed to the different ionotropic gelation mechanisms proposed for alginate and carrageenan [33]. Alginate is commonly crosslinked with Ca^2+^ by ionic inter-chain bonding, while potassium interacts with carrageenan creating only an intermolecular glue-like effect through the electrostatic attraction with the sulfate ester groups of carrageenan. This glue-like effect leads to the formation of matrices with weak entanglement, which means that higher potassium levels (≥0.3 M) could be required to produce more stable carrageenan gels. However, these high potassium levels may be undesirable for medical applications as they may cause arrhythmia and severe muscle weakness due to hyperkalemia, such as previously reported by Poncelet et al. [32], who also confirmed that in the presence of low concentrations of crosslinking solution (Ca^2+^ for alginate and K^+^ for carrageenan), alginate alone forms relatively more stable hydrogels than carrageenan alone [32]. 

### 3.3. Particle Size Analysis

The average particle size of the curcumin-loaded chitosan nanoparticles was found to be 480 ± 70 nm as determined by SEM and quantified by using the ImageJ software. The size of all the developed alginate-carrageenan hydrogel microparticles formulations was measured using a digital micrometer (General Ultra-Tech). As seen in Table 1, the freeze-dried microparticles showed relatively larger geometric sizes when compared with the air-dried microparticles. It can also be noted that regardless of the drying method, the formulations containing carrageenan resulted in larger microparticles, especially the formulations containing higher contents of carrageenan (FD3 and AD3), as compared with the formulations containing alginate only (FD1 and AD1). This may be attributed to the strong ability of the sodium alginate to form crosslinked networks upon interaction with the Ca^2+^ as compared to the crosslinking ability of the carrageenan. Consequently, increasing the carrageenan content in the developed microparticles led to a less compact structure and larger size.

### 3.4. Morphology of the Hydrogel Microparticles

**Figure 2:**
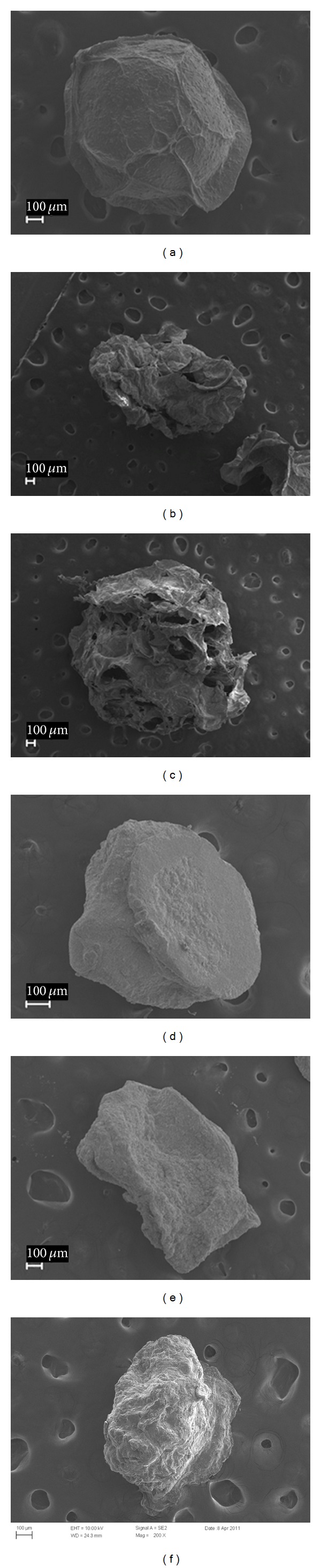
Scanning electron micrographs of alginate-carrageenan freeze-dried hydrogel microparticles (a) FD1, (b) FD2, (c) FD3, and the corresponding air-dried hydrogel microparticles (d) AD1, (e) AD2, and (f) AD3.

The SEM micrographs of microparticles containing different alginate-carrageenan ratios are shown in Figure 2. According to Figures 2(a) and 2(d), the microparticles containing alginate were smoother and more spherical compared to the microparticles including alginate and carrageenan. This effect can be attributed to the higher crosslinking ability of alginate relative to carrageenan. This crosslinking takes place instantaneously upon dropping sodium alginate into the crosslinking solution (Ca^2+^) leading to geometrically stable particles. In the case of formulations containing carrageenan (Figures 2(b), 2(c), 2(e), and 2(f)), the microparticles were less spherical, and with rough and folded surfaces. Moreover, large pores and surface cavities were observed when increasing the ratio of carrageenan in freeze-dried microparticles (Figures 2(b) and 2(c)), while air-dried microparticles were more compact (Figures 2(e) and 2(f)). This behavior could be attributed to the low concentration of K^+^ in the hardening solution, and also to the reduced crosslinking efficiency of carrageenan as compared to alginate, which led to the production of nonspherical and less physically stable microparticles. 

### 3.5. Swelling Study of Alginate-Carrageenan Microparticles

The swelling behavior of both freeze-dried and air-dried alginate-carrageenan hydrogel microparticles was studied by immersing the microparticles in simulated gastric (pH 2.1, 1 and 2 h incubation) and simulated intestinal fluids (pH 7.4: 3 up to 24 h incubation). 

**Figure 3:**
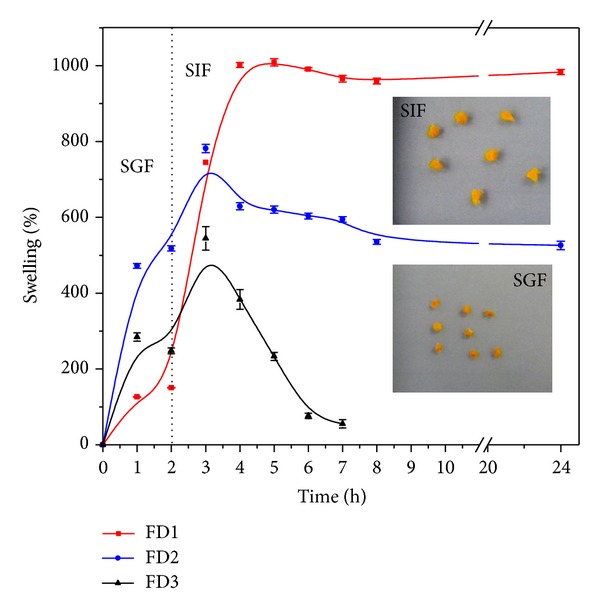
Swelling profiles of freeze-dried alginate-carrageenan microparticles of different compositions in simulated gastrointestinal fluids (SGF: pH 2.1 and SIF: 7.4) at 37°C.

**Figure 4:**
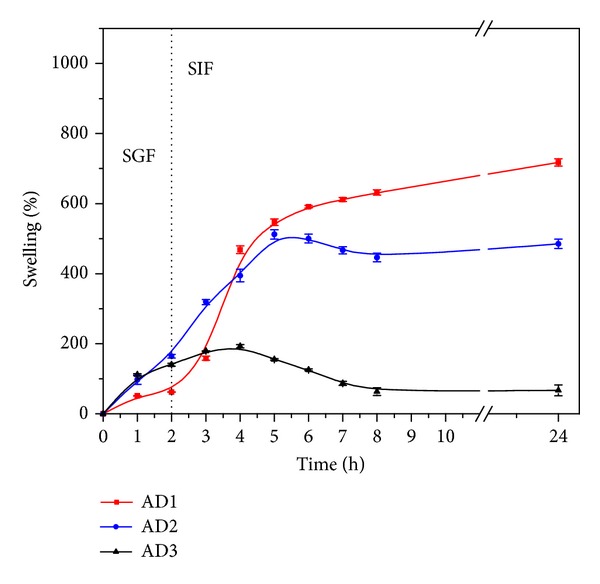
Swelling profiles of air-dried alginate-carrageenan microparticles of different compositions in simulated gastrointestinal fluids (SGF: pH 2.1 and SIF: 7.4) at 37°C.

Figures 3 and 4 compare the swelling behavior of the freeze-dried samples with the air-dried samples under similar conditions, respectively. For instance, the highest swelling values reached for the freeze-dried samples: FD1, FD2, and FD3 formulations were 1008 ± 9.3, 781 ± 11, and 544 ± 30% over their original values, respectively, whereas, the corresponding values for the air-dried samples: AD1, AD2, and AD3 were 717.9 ± 10.53%, 512.3 ± 13.38%, and 192.60 ± 4.95, respectively. The swelling values of the freeze-dried samples areattributed to the presence of pores or cavities, which facilitated the internalization of swelling fluid into the matrices. On the other hand, the compact structure of the air-dried formulations hindered the diffusion of the swelling fluid into the hydrogel particles and consequently led the attainment of relatively lower swelling values at equilibrium. 

As can be noted from Figures 3 and 4, both freeze-dried and air-dried microparticles showed different swelling behaviors in the simulated gastric fluid (SGF, pH 2.1) and in the simulated intestinal fluid (SIF, pH 7.4). At pH 2.1 (first 2 hours), all hydrogel microparticles showed a lower swelling extent as a consequence of the formation of hydrogen bonds between the polymer carboxylic and OH groups which limits their swelling, as previously reported by El-Sherbiny [41]. In contrast, at pH 7.4, the same microparticles attained higher swelling values. This behavior is attributed to the ionization of the free alginate's carboxylic groups into carboxylates (COO^−^) in the intestinal fluid (pH 7.4), which consequently generates repulsive forces between them and leads to higher swelling values [41]. The higher swelling in the intestinal fluid enhances with increasing the alginate content in both freeze- and air-dried hydrogel microparticles as seen in Figures 3 and 4. This is clearly observed in the formulations FD1 and AD1, which are composed of 100% alginate.

From Figures 3 and 4, it can also be noted that at both SGF (pH 2.1) and SIF (pH 7.4), the freeze-dried nano-microparticles attained relatively higher swelling values at equilibrium as compared to the air-dried particles. This may be due to the relatively high porosity of the freeze-dried particles as compared to the compact structure of the corresponding air-dried particles [42]. This porosity in case of the freeze-dried particles is attributed in particular to the relatively high speed of solvent sublimation in presence of the freeze dryer high vacuum, while in the case of air-dried samples the solvent evaporation occurs very slowly leading to a more compact structure of the hydrogel particles [42]. The preserved porosity of the freeze-dried hydrogel matrices facilitates the entrance of swelling fluid into the hydrogel and consequently causes higher equilibrium swelling values.

### 3.6. Curcumin Release Study

**Figure 5:**
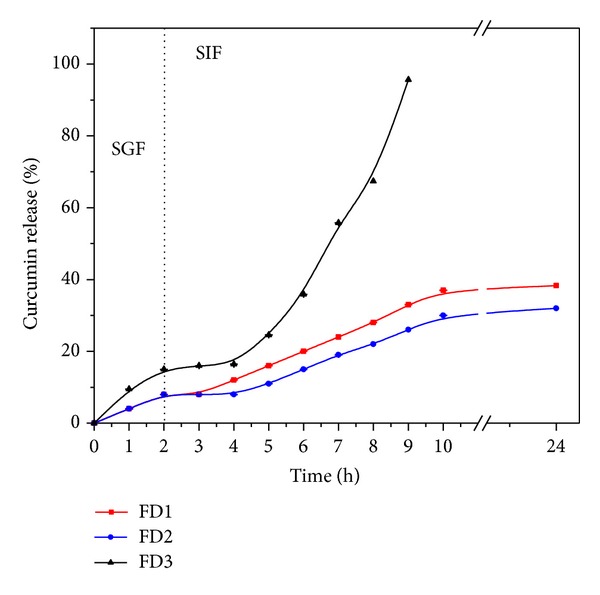
pH dependent release patterns of curcumin from freeze-dried alginate-carrageenan microparticles with different composition in simulated gastrointestinal fluids (SGF: pH 2.1 and SIF: 7.4) at 37°C.

**Figure 6:**
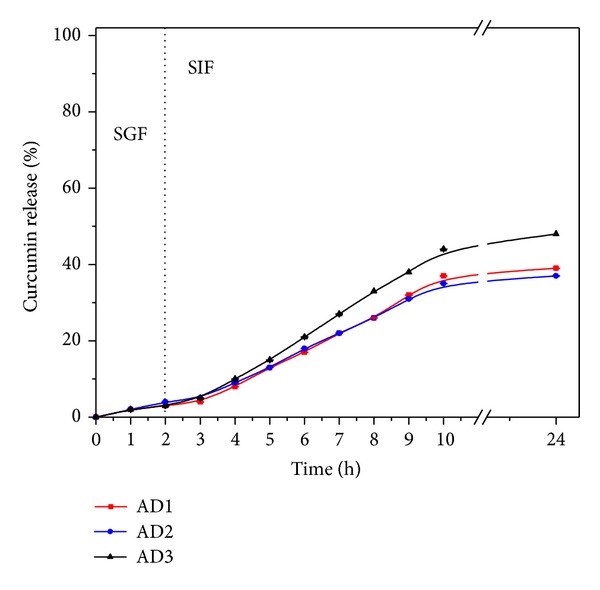
pH dependent release patterns of curcumin from air-dried alginate-carrageenan microparticles with different composition in simulated gastrointestinal fluids (SGF: pH 2.1 and SIF: 7.4) at 37°C.

The *in vitro* cumulative release profiles of curcumin from the developed alginate-carrageenan microparticles were investigated using both simulated gastric (pH 2.1) and intestinal solutions (pH 7.4) at 37°C until 24 h were completed. As shown in Figures 5 and 6, the curcumin release was higher at SIF (pH 7.4) than in SGF (pH 2.1) for both freeze-dried and air-dried microparticles following the same behavior as their swelling described in Section 3.5. 

In acidic conditions (pH 2.1), the curcumin released from either freeze-dried or air-dried microparticles was around 5 to 15% in all cases (see Figures 5 and 6). This suggests that most of the curcumin in the developed microparticles would be available to be absorbed within the intestinal tract and protected from the harsh gastric fluid. No difference was found in the curcumin release from all microparticles during the first two hours in the gastric fluid, except for FD3 formulation (Figure 5). However, when the microparticles were transferred to the SIF (pH 7.4), a slight and gradual increase in the curcumin release was observed in all formulations. This higher curcumin release could be related to the increase in swelling as alginate's carboxylic groups are ionized as described earlier.

In the microparticles formulation based on alginate alone, the percentage of curcumin released was found to be around 38% and 39% for FD1 and AD1, respectively, which is consistent with their expected high crosslinking. Formulations FD2 and AD2 showed similar behavior. On the other hand, the formulations FD3 and AD3 containing same proportion (50 : 50) of alginate and carrageenan showed the highest curcumin release percentages (96% and 48%, resp.), since the matrices were built on weak entanglements driven by the presences of carrageenan. In the particular case of the FD3 sample, a very noticeable and high percentage of curcumin was released. This high release can be attributed to the presence of holes on the surface of FD3 sample (Figure 2(c)), which ease the diffusion of the release medium into the microparticles, compared to AD1, AD2, and AD3 (Figure 2), which formed more compact microparticles due to the air-drying method. FD1 (100% alginate) and FD2 (70% alginate) formulations showed similar release patterns due to the higher content of alginate, which formed stable microparticles compared to FD3 (50% alginate). Also, it was noted that the FD3 microparticles were completely eroded after 7 h in simulated gastrointestinal fluid, while the other formulations were progressively degraded over time. This reveals that the amount of carrageenan played an important role in controlling drug release. Based on these findings we are assuming that the release profile in FD3 was controlled by the degradation of the hydrogel matrix rather than its swelling behavior.

## 4. Conclusion

This study described the development and evaluation of novel natural polymers-based nano-microparticulate system to improve the dissolution of curcumin under gastrointestinal conditions. The encapsulation of curcumin into chitosan nanoparticles showed significant enhancement of curcumin dissolution in aqueous media. A high percentage (over 95%) of the loaded curcumin was released after 7 h incubation in a pH 7.4 buffer solution upon using freeze-dried microparticles with alginate/carrageenan ratio of 50 : 50. It was found that carrageenan plays an important role in improving the release pattern of curcumin from the developed hydrogel matrices as higher release was observed in formulations with higher content of carrageenan. The study also demonstrated that some of the developed nano-microparticulate formulations (such as FD3) are very promising and need to be further investigated *in vivo*.

In conclusion, by taking advantage of the characteristics of natural polymers such as chitosan, alginate, and carrageenan, the drug release can be modulated by taking into consideration the ability of the polymers to form strong or weak hydrogel networks.

## Figures and Tables

**Table 1:** Composition of the developed alginate-carrageenan hydrogel microparticles encapsulating curcumin-loaded chitosan nanoparticles and its influence on the particle size.

Formulation code	Drying	Curcumin-loaded chitosan particles (mL)	Alginate (w%)	Carrageenan (w%)	CaCl_2_ (%)	KCl (%)	Average geometric size (*μ*m) ± SD
F1	FD	20	100	0	3	3	1034 ± 0.1
	AD	20	100	0	3	3	831 ± 0.1

F2	FD	20	70	30	3	3	1210 ± 0.2
	AD	20	70	30	3	3	1026 ± 0.1

F3	FD	20	50	50	3	3	2064 ± 0.2
	AD	20	50	50	3	3	1171 ± 0.3

FD: freeze-dried, AD: air-dried.
